# Development of a population pharmacokinetic/pharmacodynamic model for various oral paclitaxel formulations co-administered with ritonavir and thrombospondin-1 based on data from early phase clinical studies

**DOI:** 10.1007/s00280-022-04445-z

**Published:** 2022-07-07

**Authors:** Maarten van Eijk, Huixin Yu, Emilia Sawicki, Vincent A. de Weger, Bastiaan Nuijen, Thomas P. C. Dorlo, Jos H. Beijnen, Alwin D. R. Huitema

**Affiliations:** 1 Department of Pharmacy & Pharmacology, Netherlands Cancer Institute, Antoni van Leeuwenhoek, Plesmanlaan 121, 1066 CX Amsterdam, The Netherlands; 2 Modra Pharmaceuticals Holding B.V., Amsterdam, The Netherlands; 3 Department of Clinical Pharmacology, Netherlands Cancer Institute, Antoni van Leeuwenhoek, Amsterdam, The Netherlands; 4 Utrecht Institute of Pharmaceutical Sciences, Utrecht University, Utrecht, The Netherlands; 5 Department of Clinical Pharmacy, University Medical Center Utrecht, Utrecht University, Utrecht, The Netherlands; 6 Department of Pharmacology, Princess Máxima Center for Pediatric Oncology, Utrecht, The Netherlands

**Keywords:** Oral paclitaxel, Ritonavir, CYP3A4, Population pharmacokinetics, Thrombospondin-1, Low-dose metronomic therapy

## Abstract

**Purpose:**

Orally administered paclitaxel offers increased patient convenience while providing a method to prolong exposure without long continuous, or repeated, intravenous infusions. The oral bioavailability of paclitaxel is improved through co-administration with ritonavir and application of a suitable pharmaceutical formulation, which addresses the dissolution-limited absorption of paclitaxel. We aimed to characterize the pharmacokinetics of different paclitaxel formulations, co-administered with ritonavir, and to investigate a pharmacodynamic relationship between low-dose metronomic (LDM) treatment with oral paclitaxel and the anti-angiogenic marker thrombospondin-1 (TSP-1).

**Methods:**

Fifty-eight patients treated with different oral paclitaxel formulations were included for pharmacokinetic analysis. Pharmacodynamic data was available for 36 patients. All population pharmacokinetic/pharmacodynamic modelling was performed using non-linear mixed-effects modelling.

**Results:**

A pharmacokinetic model consisting of gut, liver, central, and peripheral compartments was developed for paclitaxel. The gastrointestinal absorption rate was modelled with a Weibull function. Relative gut bioavailabilities of the tablet and capsule formulations, as fractions of the gut bioavailability of the drinking solution, were estimated to be 0.97 (95%CI: 0.67–1.33) and 0.46 (95%CI: 0.34–0.61), respectively. The pharmacokinetic/pharmacodynamic relationship between paclitaxel and TSP-1 was modelled using a turnover model with paclitaxel plasma concentrations driving an increase in TSP-1 formation rate following an E_max_ relationship with an EC_50_ of 284 ng/mL (95%CI: 122–724).

**Conclusion:**

The developed pharmacokinetic model adequately described the paclitaxel plasma concentrations for the different oral formulations co-administered with ritonavir. This model, and the established pharmacokinetic/pharmacodynamic relationship with TSP-1, may facilitate future development of oral paclitaxel.

**Supplementary Information:**

The online version contains supplementary material available at 10.1007/s00280-022-04445-z.

## Introduction

Paclitaxel is a microtubule stabilizing agent that is used in the treatment of various malignancies such as breast cancer, non-small-cell lung cancer (NSCLC), and ovarian cancer [1]. It is intravenously (IV) administered in either a weekly or a 3-weekly schedule as single-agent treatment or in combination with other cytotoxic agents [2]. In vitro, the cytotoxic effect of paclitaxel is positively related to the duration of exposure [3]. Clinically, it has been demonstrated that in ovarian and breast cancer patients, treated with single-agent IV paclitaxel, the time during which paclitaxel plasma concentrations exceed 0.05 µmol/L (T_C>0.05_, 42.7 ng/mL) or 0.1 µmol/L (T_C>0.1,_ 85.4 ng/mL) was predictive of myelosuppression [4, 5]. Whereas when combined with carboplatin in the treatment of NSCLC, the paclitaxel T_C>0.1_ was associated with improved survival [6]. Similarly, in ovarian cancer patients treated with this combination, T_C>0.05_ has shown to be both a predictor of clinical outcome and severe neutropenia [7]. Prolonged exposure above certain threshold levels can potentially be easier attained using a low-dose metronomic (LDM) chemotherapy regimen, i.e., frequent administration of a cytotoxic drug at a relatively low dose. In addition, previous studies have shown that LDM chemotherapy with paclitaxel possesses anti-cancer activity by inhibiting angiogenesis with limited side effects [8–10].

Metronomic chemotherapy with IV paclitaxel is not feasible due to the significant patient burden associated with daily intravenous administration. Furthermore, the paclitaxel IV formulation contains ethanol and polyethoxylated castor oil (Cremophor EL), the latter of which may induce hypersensitivity reactions and neuropathy [11]. An oral formulation may therefore facilitate the successful implementation of LDM chemotherapy with paclitaxel.

Unfortunately, paclitaxel has a low oral bioavailability. This is due to its low aqueous solubility, high affinity for the P-glycoprotein (P-gp) efflux transporter, and metabolism by cytochrome P450 (CYP)3A4 and CYP2C8 [12–15]. An oral formulation has been developed which addresses some of these issues. Solubility could be enhanced by formulating paclitaxel as either a freeze-dried or a spray dried amorphous solid dispersion (ASD) capsule or tablet formulation [16, 17]. Additionally, the bioavailability was boosted by inhibiting gastrointestinal and hepatic CYP3A4 and P-gp through co-administration with the potent CYP3A4 inhibitor, and moderate P-gp inhibitor, ritonavir [18, 19]. Several early phase clinical studies have shown that clinically relevant paclitaxel plasma concentrations could be achieved using these strategies [20, 21].

Preclinical studies have raised the hypothesis that the anti-angiogenic effects of paclitaxel LDM treatment are brought about by upregulation of the expression of thrombospondin-1 (TSP-1) [22–24]. TSP-1 is a component of the extracellular matrix which can be found in the circulation and which is thought to modulate angiogenesis through inhibition of nitric oxide-mediated signaling in endothelial cells, vascular smooth muscle cells, and platelets [8]. This effect is attributed to binding of TSP-1 to the CD36 and CD47 cell surface receptors [25]. However, TSP-1 is a multifaceted protein with a wide variety of ligands which complicates its translational potential [25]. Moreover, clinical studies have yet to demonstrate beneficial effects of TSP-1 upregulation or its use as a potential biomarker in patients treated with LDM [26]. The clinical relevance of TSP-1 upregulation during LDM treatment with paclitaxel is therefore still unclear.

The objective of this study was to develop a model that describes the complex population pharmacokinetics (PK) of different formulations of orally administered paclitaxel co-administered with ritonavir, and subsequently use this developed model in simulations to compare PK characteristics of oral and IV paclitaxel. Finally, we sought to model the potential relationship between the PK of LDM treatment with paclitaxel and its pharmacodynamic (PD) effect on TSP-1.

## Methods

### Oral paclitaxel formulations

Three different oral paclitaxel formulations were previously investigated in various clinical studies [16, 20, 27].

- Registered IV formulations of paclitaxel (6 mg/mL dissolved in ethanol and Cremophor EL1:1 w/v, Mayne Pharma, Melbourne Australia or Paxene, Norton Healthcare Ltd., London, United Kingdom) were used as a drinking solution [16, 27].
- ModraPac capsules, the production of which was previously described by Moes et al. [16]. Briefly, ModraPac capsules consist of a freeze-dried ASD of 10 mg paclitaxel combined with povidone K30 and sodium dodecyl sulphate (SDS) in a weight ratio of 1/9/1 (w/w/w). This ASD is blended with lactose and anhydrous colloidal silicon dioxide and filled into gelatin capsules.
- ModraPac tablets, the production of which has previously been described by Sawicki et al. [17]. In short, the ASD (paclitaxel–povidone K30–SDS 1:9:1 w/w/w) was prepared using spray drying and subsequently tablets were manufactured by addition of lactose monohydrate (SuperTab 30 GR^®^, 75% of tablet weight), croscarmellose sodium (3% of tablet weight), anhydrous colloidal silicon dioxide (1% of tablet weight), and magnesium stearate (1% of tablet weight).

### Pharmacokinetic data

The PK data originated from three clinical studies which included a total of 58 patients [16, 20, 27]. A summary of applied doses, dosing, and sampling times is shown in Supplementary Table 1.

- Study 1 was a proof-of-concept study in which the paclitaxel drinking solution was co-administered with ritonavir [27]. The drinking solution of paclitaxel was administered as a single 100 mg dose in 17 patients, with 100 or 200 mg ritonavir (Norvir^®^; Abbott Laboratories Ltd., Illinois, USA) administered 30 min prior to paclitaxel [27].
- Study 2 was a randomized proof-of-concept study in which four patients received both the ModraPac capsule formulation and the drinking solution at a dose of 30 mg once weekly co-administered with 100 mg ritonavir (Norvir^®^; Abbott Laboratories Ltd., Illinois, USA) 30 min prior to paclitaxel. Treatment was administered over the course of two subsequent weeks. Patients were randomized to receive either the drinking solution in the first week and the ModraPac capsule formulation in the second or vice versa [16].
- Study 3 was a phase I dose escalation LDM study of oral paclitaxel in combination with ritonavir which included 37 patients. ModraPac capsules or ModraPac tablets were given to patients twice daily together with ritonavir (Norvir^®^; Abbott Laboratories Ltd., Illinois, USA) with a 7-h interval. The daily doses studied for ModraPac capsule formulation included 5, 10, 15, 20, 30, and 40 mg; and for the ModraPac tablet formulation 40, 50, and 60 mg. Ritonavir was administered at a daily dose of 200 mg in all dose levels. This study was registered in the Dutch trial registry (NTR3632) and European Union Drug Regulating Authorities Clinical Trials (EudraCT) Database (2010-021,525-13) [20].

### Pharmacodynamic data

Measurement of TSP-1 was only implemented in the study with LDM dosing. Sample preparation and analysis have been described in detail in the original publication [20]. PD data were available for 36 out of 37 patients; all treated at different dose levels. Samples were obtained on days 1, 2, and 8 in the first cycle; day 1 of cycle 2; day 1 of cycle 3; every 6 weeks thereafter, and at disease progression. Since TSP-1 is taken up from the circulation by platelets, TSP-1 concentrations were quantified relative to platelet counts (ng/mL/10^6^ platelets) [20, 28].

### Structural model development

#### Pharmacokinetic model

A previously developed population PK model for orally administered ritonavir was applied to our current PK data. In this model, ritonavir PK were described using a two-compartment model with a first-order elimination process. The absorption was modelled using an inverse Gaussian density input function [29].

For paclitaxel, various numbers of PK compartments were explored and different absorption models were screened. Clearance and first-pass effect were modelled using the assumption of a well-stirred liver model [30]. In this, an inhibitory maximum effect model was used to calculate paclitaxel hepatic clearance as a function of the uninhibited intrinsic clearance (CL_int0_), the ritonavir plasma concentration (C_ritonavir_), the estimated maximum effect (I_max_), and the inhibition constant (KI) of CYP3A4 by ritonavir (Eq. 1.) Paclitaxel extraction ratio (*E*_*H*_) and hepatic bioavailability (*F*_*H*_) were defined as in Eq. 2 and Eq. 3. Here, hepatic blood flow (*Q*_*H*_*)* was fixed at a value of 80 L·h^−1^ [31]. Liver volume (*V*_*H*_*)* was fixed to 1 L, which is close to values previously reported [32]. Since only total paclitaxel concentrations were available, a literature estimate for the fraction unbound (*fu*) paclitaxel of 13% was used for the calculation of the hepatic extraction ratio [33]. The effect of the type of oral paclitaxel formulation was investigated as covariate on relative gut bioavailability (*rF*_*gut*_), which thus represents a composite estimate for the relative fraction that circumvents gastrointestinal metabolism and efflux by transporters as well as the effect of each formulation on gut bioavailability. In addition, the effect of the different oral paclitaxel formulations on absorption characteristics was investigated

1 $$CL_{{\text{int}}} \left( t \right) = CL_{{{\text{int}} 0}} - \frac{{I_{\max } \cdot C_{ritonavir} \left( t \right)}}{{KI + C_{ritonavir} \left( t \right)}}$$

2 $$E_{H} \left( t \right) = \frac{{CL_{{\text{int}}} \left( t \right) \cdot fu}}{{Q_{H} + CL_{{\text{int}}} \left( t \right) \cdot fu}}$$

3 $$F_{H} \left( t \right) = 1 - E_{H} \left( t \right).$$

#### TSP-1 PD model

**Fig. 1:**
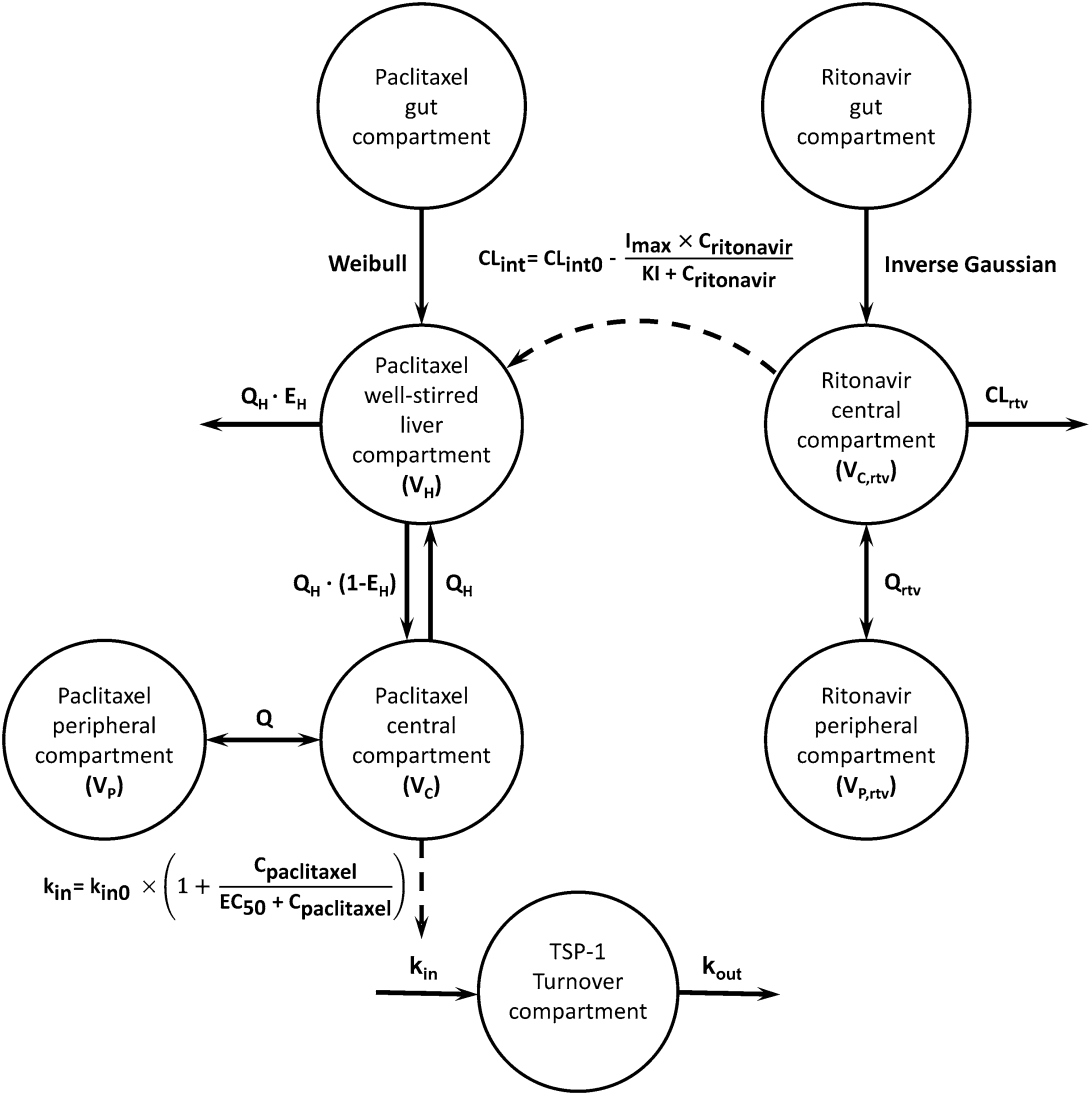
Schematic structure of the developed PK/PD model for oral paclitaxel, co-administered with ritonavir, and TSP-1. *CL*_*rtv*_ clearance, *CL*_*int*_ paclitaxel intrinsic clearance, *CL*_*int0*_ paclitaxel uninhibited intrinsic clearance, *C*_*pac*_ paclitaxel plasma concentration, *C*_*rtv*_ ritonavir plasma concentration, *E*_*H*_ hepatic extraction ratio, *EC*_*50*_ concentration at which E is 50% of E_max_, *I*_*max*_ maximum inhibitory effect, *KI* inhibition factor at which 50% of I_max_ is produced, *k*_*in*_ input rate constant turnover compartment, *k*_*in0*_ baseline input rate constant turnover compartment, *k*_*out*_ output rate constant turnover compartment, *Q* paclitaxel inter-compartment distribution, *Q*_*H*_ hepatic blood flow, *Q*_*rtv*_ ritonavir inter-compartment distribution, *TSP-1* Thrombospondin-1, *V*_*C*_ paclitaxel central volume of distribution, *V*_*H*_ paclitaxel hepatic volume of distribution, *V*_*P*_ paclitaxel peripheral volume of distribution, *V*_*C,rtv*_ ritonavir central volume of distribution, *V*_*P,rtv*_ ritonavir peripheral volume of distribution

Based on physiological knowledge of TSP-1, we considered a turnover model to be the most appropriate starting point for modelling the PD effect [34]. In this model, TSP-1 concentrations (C_TSP-1_) were assumed to be at steady state at baseline with a zero-order formation rate (k_in_) and a first-order elimination rate (k_out_) (Fig. 1, Eq. 4). The first-order elimination rate k_out_ was defined by the inverse of the TSP-1 turnover time (Eq. 5). Paclitaxel plasma concentrations (C_paclitaxel_) were assumed to drive the drug effect, increasing the TSP-1 formation rate following a sigmoidal relationship (Fig. 1, Eq. 6). The ritonavir and paclitaxel PK and TSP-1 PD were modelled sequentially using the Population PK Parameters (PPP) approach, as described by Zhang et al. [35]

4 $$\frac{{dC_{{TSP{-}1}} }}{dt} = k_{in} - k_{out} \cdot C_{{TSP{-}1}}$$

5 $$k_{out} = \frac{1}{Turnover}$$

6 $$k_{in} = k_{in0} \cdot \left( {1 + \frac{{C_{paclitaxel} }}{{EC_{50} + C_{paclitaxel} }}} \right).$$

### Statistical model development

Both between-subject variability (BSV) and between-occasion variability (BOV) were modelled using an exponential distribution according to Eq. 7. BOV was only evaluated in patients who received doses on multiple occasions. Each dose administration was considered an occasion

7 $$P_{i} = P \cdot \exp \left( {\eta_{i,BSV} + \eta_{i,BOV} } \right),$$

where *P*_*i*_ represents the individual parameter estimate for individual *i*, *P* represents the typical population parameter estimate, and *η*_*i*_ either BSV or BOV effect distributed following *N (0, ω*^*2*^*)*.

The residual error was modelled using proportional error models for both paclitaxel and TSP-1, respectively (Eq. 8)

8 $$C_{obs,ij} = C_{pred,ij} \cdot \left( {1 + \varepsilon_{p,ij} } \right),$$

where *C*_*obs,ij*_ or *C*_*pred,ij*_ represents, for the *i*th subject and the *j*th measurement, the observation or prediction. The proportional error *ε*_*p,ij*_ was assumed distributed following *N (0, σ*^*2*^*)*.

### Model evaluation

Acceptable models were required to achieve a successful minimization and covariance step. Different models were evaluated based on the stability, plausibility and precision of parameter estimates, goodness-of-fit (GOF) plots, and drop in objective function value (OFV) with significance level of p < 0.01 (degree of freedom (df) = 1, dOFV > 6.63; df = 2, dOFV > 9.21) for hierarchical models. Additionally, due to a wide range in dose levels for which PK data were available, a prediction-corrected visual predictive check (VPC) was performed (*n* = 1000). A sampling importance resampling (SIR) algorithm was implemented to evaluate the uncertainty of the final parameter estimates in the oral paclitaxel PK–PD model [36].

### Simulations

We performed simulations to compare the PK characteristics (C_max_, AUC_0-504 h_, T_C>0.05_) of two IV schedules and LDM treatment with the oral paclitaxel tablet formulation. The population parameter estimates and model for IV paclitaxel presented by Joerger et al. were used to simulate plasma concentrations for the IV schedules [37]. A 3-week dosing schedule at 175 mg/m^2^ (BSA = 1.8 m^2^) with 3-h infusion [37], and a weekly dosing schedule at 80 mg/m^2^ (BSA = 1.8 m^2^) with 1-h infusion [38, 39] were simulated. Since the tablet formulation was considered the most suitable for further clinical development, the PK of the ModraPac tablet formulation was simulated continuously for 3 weeks at a dose level of 20 mg twice daily, co-administered with ritonavir (100 mg, twice daily) with a 7-h interval between paclitaxel doses; which is the recommended phase II dose (RP2D) for the tablet formulation based on the phase I dose finding study with LDM paclitaxel [20].

In addition, PK simulations based on population parameter estimates were performed to compare the PK characteristics (T_max_, C_max_ AUC_0-24 h_) of both paclitaxel ASD formulations. Plasma concentrations were simulated based on population parameter estimates for the ModraPac capsule and tablet formulation at the RP2D.

### Software

Model estimations and simulations were performed using non-linear mixed-effects modelling software (NONMEM, version 7.3, ICON Development Solutions, Ellicott City, MD, USA) together with a gfortran compiler [40]. Models were fit with the first-order conditional estimation method with the interaction option (FOCE-I). Pirana (version 2.9.9) was used as graphical interface [41], and R (version 4.0.3) was used for pre-processing of the data, plotting, and model simulation [42]. In addition, the NONMEM toolkit PsN [43], and the R-packages Xpose [44] and deSolve [45] were used.

## Results

### Model development

#### Oral paclitaxel pharmacokinetic model

The final PK/PD model structure is shown in Fig. 1. Ritonavir PK was well described using the previously developed model [29]. Individual Bayesian parameter estimates for ritonavir, generated using this model, were used as input for the development of the paclitaxel PK model. For patients where no ritonavir plasma concentrations were sampled, ritonavir PK was incorporated using fixed effects parameter estimates.

The parameter estimates for the oral paclitaxel PK model are presented in Table 1. The paclitaxel PK model consisted of gut, liver, central, and peripheral compartments. The intrinsic clearance (*CL*_*int0*_*)* in the well-stirred liver model was estimated at 746 L/h (95% CI: 585–937) with the ritonavir inhibitory effect factor (KI) estimated as 375 ng/mL (95% CI: 135–906).

**Table 1:** Parameter estimates for oral paclitaxel in the final PK model

Parameters	Units	Estimate	95% CI	Shrinkage (%)
Population parameters				
ALPHA_1st daily dose_	–	1.68	1.52–1.87	–
ALPHA_2nd daily dose_	–	1.97	1.79–2.19	
BETA_drinking solution_	–	2.53	2.34–2.76	–
BETA_tablet+capsule_	–	3.57	2.99–4.52	–
CL_int0_	L/h	746	585–937	–
KI	ng/mL	375	135–906	
I_max_	L/h	570	400–776	–
Vc	L	128	105–151	–
Q	L/h	33.4	29.6–37.6	–
Vp	L	375	311–465	–
* rF*_drinking solution_	–	1 FIX		–
* rF*_tablet_	–	0.97	0.67–1.33	–
* rF*_capsule_	–	0.46	0.34–0.61	–
* rF*_2nd/1st_	–	0.59	0.48–0.74	–
Between-subject variability				
ALPHA	CV%	35.1	28.9–43.5	7
CL_int0_	CV%	25.1	17.8–36.1	24
Vc	CV%	53.8	42.2–68.8	13
* rF*_*gut*_	CV%	38.2	25.1–51.4	32
Between-occasion variability				
* rF*_gut_	CV%	45.8	35.8–59.7	
Residual unexplained variability				
σ_prop_	CV%	25.8	24.4–27.4	11

*ALPHA*_*1st daily dose*_ scale parameter in Weibull function for the first daily dose, *ALPHA*_*2nd daily dose*_ scale parameter in Weibull function for the second daily dose, *BETA*_*drinking solution*_ shape parameter in Weibull function for the drinking solution, *BETA*_*capsule/tablet*_ shape parameter in Weibull function for the capsule and tablet formulation, *CI* Confidence interval, *CL*_*int0*_ uninhibited intrinsic clearance, *CV%* coefficient of variation, *I*_*max*_ maximum inhibitory effect, *KI* inhibition factor at which 50% of I_max_ is produced; *PAC* paclitaxel, *σ*_*prop*_ proportional residual error, *Q* intercompartmental clearance, *rF*_*drinking solution*_ relative gut bioavailability of the drinking solution, *rF*_*tablet*_ relative gut bioavailability of tablet formulation, *rF*_*capsule*_ relative gut bioavailability of capsule formulation, *rF*_*2nd/1st*_ relative gut bioavailability of second dose compared to the first, *Vc* volume of distribution of central compartment, *Vp* volume of distribution of peripheral compartment

The absorption rate of paclitaxel from the gut to the liver compartment was modelled using a Weibull function. Implementation of this absorption model allowed for the modelling of a time-varying absorption rate, which more accurately represented the physiological processes of dissolution and absorption of paclitaxel from the oral formulations compared to first-order or zero-order absorption models (Eq. 9)

9 $$k_{a} \left( t \right) = \left( {\frac{BETA}{{ALPHA}}} \right) \cdot \left( \frac{t}{ALPHA} \right)^{{\left( {BETA - 1} \right)}} \cdot {\text{exp}}\left( { - \left( \frac{t}{ALPHA} \right)^{BETA} } \right).$$

In this function, ALPHA and BETA, respectively, represent the scale and the shape parameter of the Weibull function over time (*t*), with *t* defined as the time after the last dose. The Weibull absorption model was expanded further by estimating separate fixed effects for the BETA parameter for the ASD formulations and the drinking solution (BETA_drinking solution_, BETA_tablet/capsule_) and ALPHA parameter for the first and second daily dose (ALPHA_1st daily dose,_ ALPHA_2nd daily dose_). The addition of these covariate effects allowed for a better description of the variable absorption rate curve for the drinking solution and the ASD formulations and for each of the daily dosing occasions.

The estimates for the shape parameter in the Weibull function, BETA_drinking solution_ 2.53 (95% CI: 2.34–2.76), BETA_tablet/capsule_ 3.57 (95% CI: 2.99–4.52), indicate a faster initial absorption rate for the drinking solution compared to the ASD formulations (Supplementary figure S1). While, estimates for ALPHA_1st daily dose_ and ALPHA_2nd daily dose_ (1.68, 95% CI: 1.52–1.87 and 1.97, 95% CI: 1.79–2.19) signify a shift in the absorption profile of the second daily dose compared to the first daily dose for the ASD formulations (Supplementary figure S1).

The influences of the different oral paclitaxel formulations on the relative gut bioavailability (*F*_gut_) were estimated as a covariate effects (*rF*_capsule_ and *rF*_tablet_) relative to the drinking solution. The parameters *rF*_tablet_ and *rF*_capsule_ were estimated to be 0.97 (95% CI: 0.67–1.33) and 0.46 (95% CI: 0.34–0.61), respectively. BSV on *rF*_gut_ was estimated to be 38.2 CV% (95% CI: 25.1–51.4). While BOV on *rF*_gut_ was 45.8 CV% (95% CI: 35.8–59.7). In addition, for patients taking oral paclitaxel in a twice daily schedule, an empirical parameter, *rF*_2nd/1st_ was introduced, which characterized differences in relative bioavailability between the first and second daily dose. The model fit was found to significantly improve after inclusion of this parameter. The value of *rF*_2nd/1st_ was estimated as 0.59 (95% CI: 0.48–0.74) indicating a decreased bioavailability of the second daily dose. The effect of the once daily 200 mg ritonavir dose that was administered in five patients as opposed to the once daily 100 mg dose, or twice daily 100 mg dose was also investigated as a covariate on relative gut bioavailability, but did not suggest a strong difference in relative gut bioavailability or improve the model fit and was therefore not included in the model.

#### TSP-1 PD model

Figure 1 schematically shows the relationship between paclitaxel PK and the TSP-1 PD. The parameter estimates for the PD model are presented in Table 2. Representative curves of TSP-1 observations, population predictions, and individual predictions for four patients treated with different doses of oral paclitaxel in an LDM schedule are shown in Supplementary figure S2. In the turnover model, upregulation of TSP-1, driven by paclitaxel plasma concentrations (C_paclitaxel_), was modelled using an E_max_ model with an estimated E_BASE_ of 43.8 ng/mL/10^6^ platelets (95% CI: 39.7–48.5) and EC_50_ of 284 ng/mL (95% CI: 122–724). The turnover time for TSP-1 was fixed to a literature value for platelet survival (9.7 days, [46]) as this parameter was not identifiable. The developed PD model was evaluated against a model with the assumption of no effect (EC_50_ fixed at 10^5^ ng/mL); which demonstrated that the model, with the drug effect of paclitaxel on TSP-1 concentrations included, had a significantly better fit of the data (dOFV = − 12.52).

**Table 2:** Parameter estimates for thrombospondin-1 in the final PD model

Parameters	Units	Estimate	95% CI	Shrinkage (%)
**Population parameters**				
EC_50_	ng/mL	284	122–724	–
E_BASE_	ng/mL/10^6 ^platelets	43.8	39.7–48.5	–
Turnover	h	233 FIX	–	–
**Between-subject variability**				
E_BASE_	CV%	28.2	22.8 – 36.6	4
**Residual unexplained variability**				
σ_prop_	CV%	13.8	12.3 – 15.8	12

### Model evaluation

**Fig. 2:**
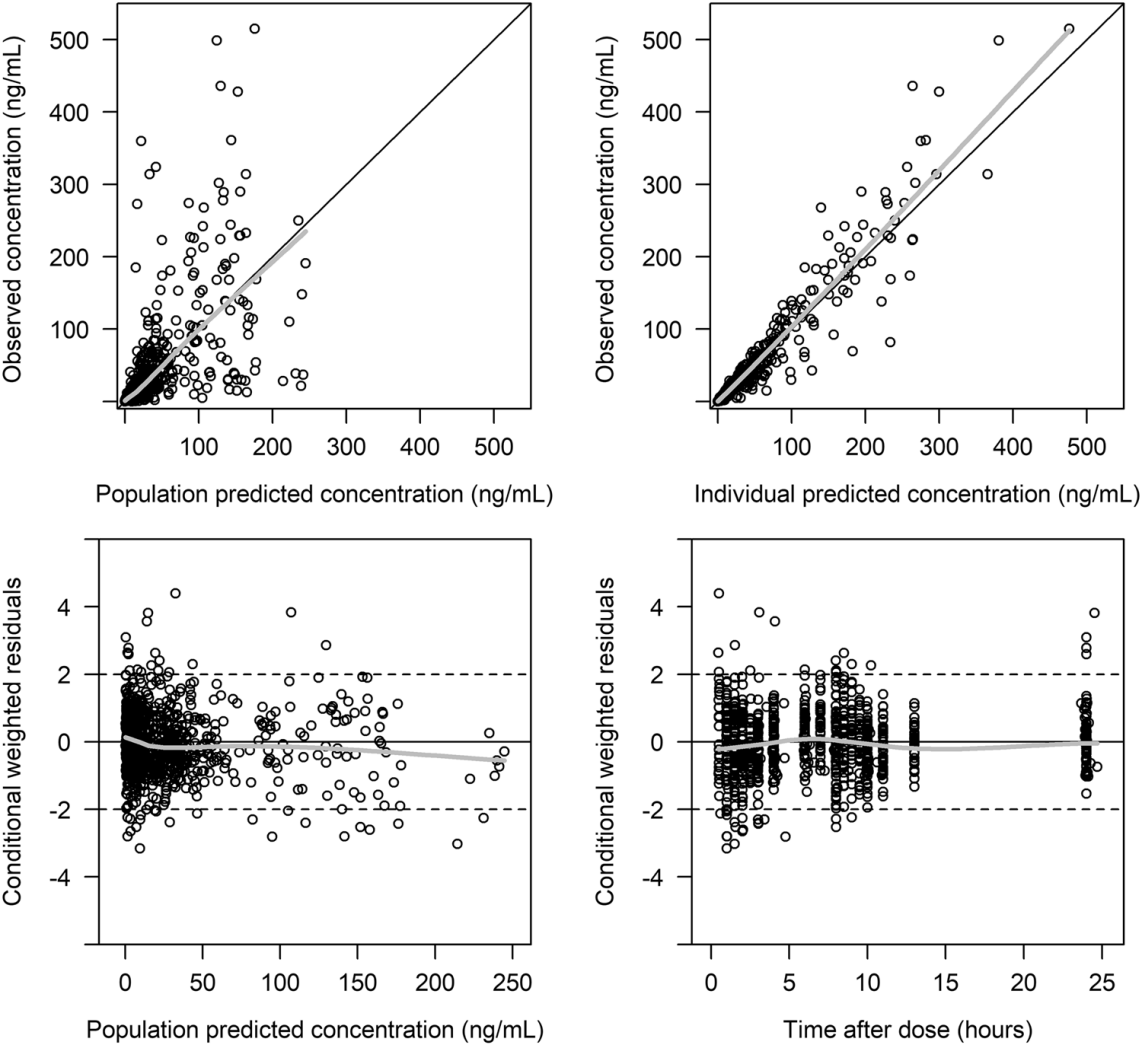
Goodness-of-fit plots for the paclitaxel PK model. The plots include observed versus population predicted concentration, observed versus individual model predicted concentration, conditional weighted residuals (CWRES) versus population predicted concentration, and CWRES versus time

**Fig. 3:**
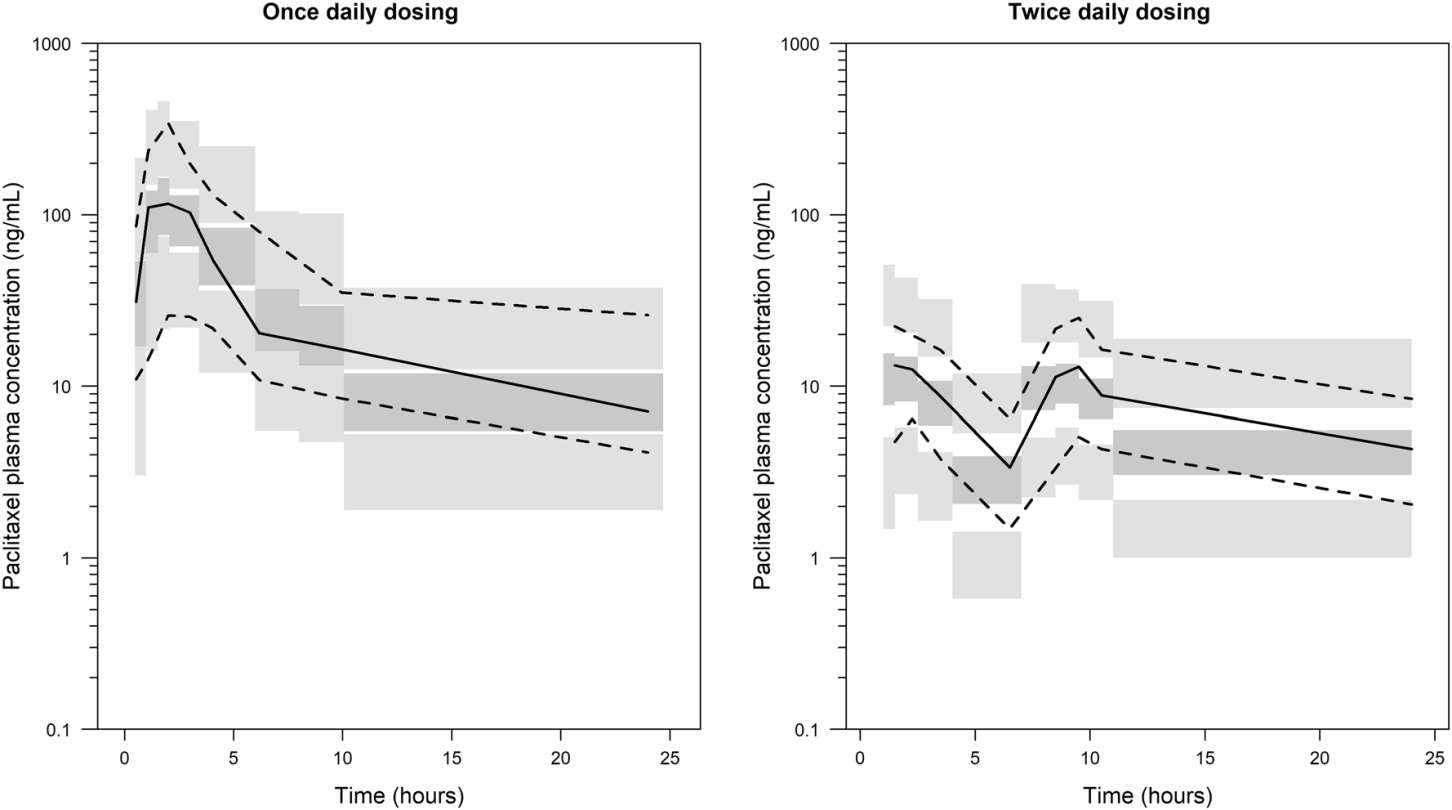
Prediction-corrected visual predictive check of paclitaxel plasma concentration for oral paclitaxel formulations stratified by schedule of administration (*n* = 1000). Solid lines and dark grey areas represent the median observed values and simulated 95% CIs. Dashed lines and light grey areas represent the 10% and 90% percentiles of the observed values and 95% CIs of the simulated percentiles

GOF plots (Fig. 2) and prediction-corrected VPC (Fig. 3) demonstrated that the developed final model adequately described the paclitaxel PK observations. There was no obvious bias of the model differentiated by study designs. Similarly, the developed PD model was found to adequately describe the TSP-1 biomarker observations (Supplementary figure S3).

### Simulations

**Fig. 4:**
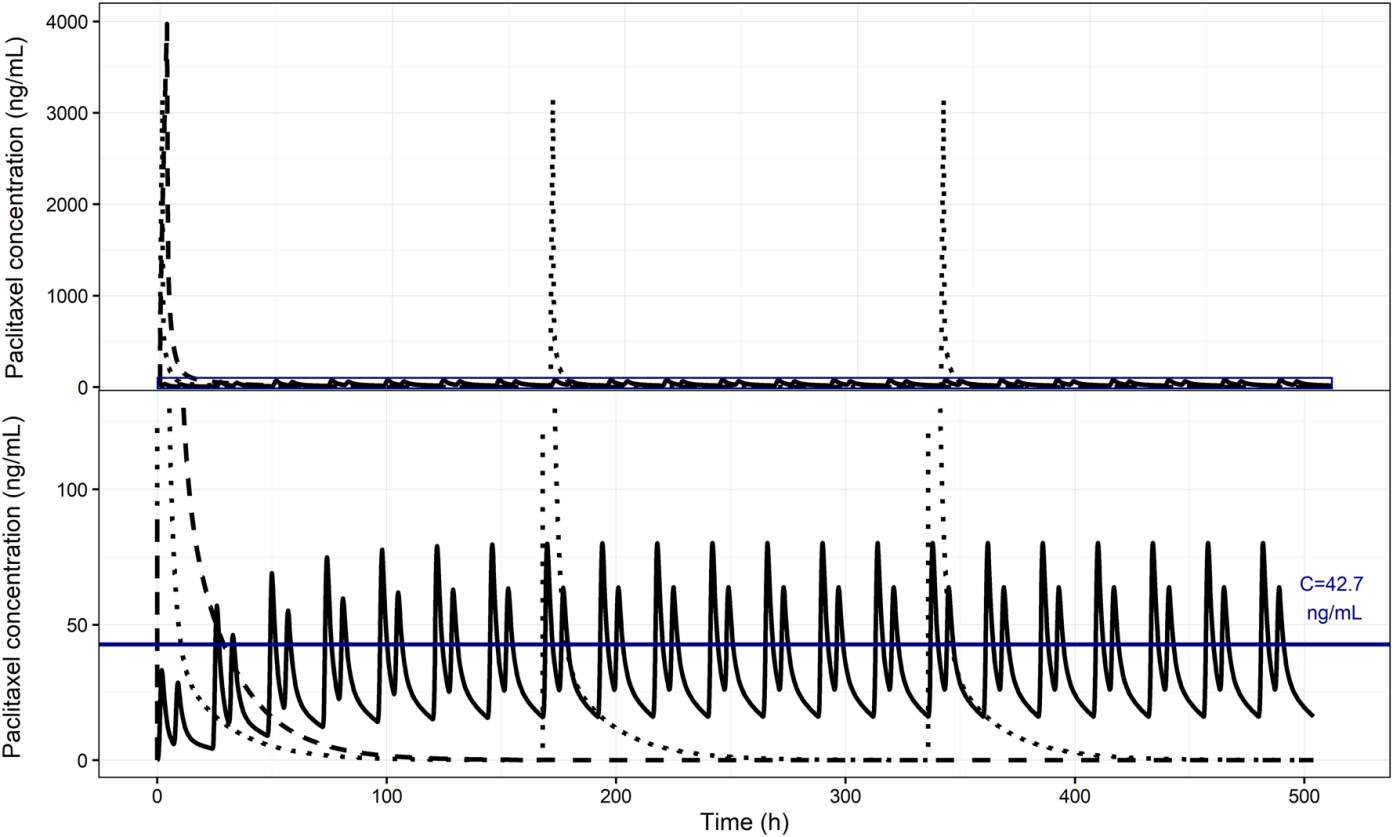
Comparison of the PK (0–504 h) between intravenous schedules of paclitaxel and the ModraPac tablet formulation co-administered with ritonavir administered twice daily. The dashed curve represents the plasma concentration of IV paclitaxel given every 3 weeks as a 3 h infusion at a dose of 175 mg/m^2^ (BSA = 1.8 m^2^). The dotted curve represents the plasma concentration of IV paclitaxel given every week as a 1 h infusion at a dose of 80 mg/m^2^ (BSA = 1.8 m^2^). The solid curve represents the paclitaxel plasma concentration of the ModraPac tablet formulation (20 mg twice daily) co-administered with ritonavir (100 mg twice daily). The upper panel shows the complete scope of the concentration–time curves. The lower panel is the zoomed rectangular area in the upper panel. The solid blue line indicates the paclitaxel plasma concentration at 42.7 ng/mL (0.05 µmol/L)

Simulations based on population parameter estimates were performed to compare PK characteristics of the IV formulation of paclitaxel and ModraPac tablet applied in different schedules (Fig. 4). The maximum concentration (C_max_) following 3-weekly infusion of the IV formulation was 3.97 µg/mL, C_max_ of IV formulation with weekly infusion was 3.16 µg/mL, and the steady-state C_max_ of ModraPac tablet formulation was 8.01 10^–2^ µg/mL. For weekly, 3-weekly IV administration and LDM with the ModraPac tablet in a 3-week period the AUC_0–504_ was 14.6 10^3^ µg h/L, 13.6 10^3^ µg h/L, and 16.0 10^3^ µg h/L, respectively. The simulated cumulative T_C>0.05_ for 3-weekly or weekly IV administration was 29.0 h or 30.8 h. For the ModraPac tablet formulation, the simulated cumulative T_C>0.05_ per 3-week interval was 115.6 h.

The simulation of paclitaxel plasma concentration over time curves based on population parameter estimates for the oral ModraPac capsule and tablet formulation (20 mg twice daily) co-administered with ritonavir (100 mg twice daily) is shown in Supplementary figure S4. The time to maximum concentration (T_max_) in the first- and second-dose intervals were 2.1 h and 2.4 h after administration, respectively. The respective areas under the concentration–time curve for 24 h (AUC_0–24 h_) of ModraPac capsule and tablet at this dose level were 131.4 µg·h/L and 275.1 µg·h/L, respectively.

## Discussion

We successfully established a PK model for three oral paclitaxel formulations co-administered with ritonavir. The final model included a central and one peripheral compartment to describe the paclitaxel plasma concentrations. As opposed to previous models for IV paclitaxel, which implemented two peripheral compartments, we found that a single peripheral compartment was sufficient to describe oral paclitaxel PK [47]. Possibly, this is due to absence of Cremophor EL in the systemic circulation, which is thought to be responsible for the non-linear PK of IV paclitaxel through entrapment of the drug in Cremophor EL micelles [48].

Several model elements were key in the adequate description of the observed PK of paclitaxel after oral administration together with ritonavir. First, the Weibull function successfully captured the variable absorption profile of oral paclitaxel. This variable absorption profile is the result of the complex absorption of paclitaxel with the influence of factors such as variability in intestinal paclitaxel dissolution and precipitation, P-gp mediated drug transport, and ritonavir co-administration. Previously, a PK model was established for another oral paclitaxel formulation (DHP107) where a similar model structure was applied [49]. Second, our model included the interaction between the CYP3A4 inhibitor ritonavir and intrinsic paclitaxel clearance, which was included in a well-stirred liver model following an inhibitory I_max_ relationship (Eq. 1). This inhibition model allowed for the quantitation of the effect of ritonavir on total intrinsic paclitaxel clearance over time (Supplementary Figure S5). A previous population PK model for oral docetaxel has been published by our group in which a general competitive inhibition model was used to describe the effect of ritonavir on hepatic clearance [29]. To model paclitaxel intrinsic clearance, we considered an inhibition model which accounts for the presence of other metabolism routes more suitable. Specifically due to the contribution of CYP2C8 to total paclitaxel clearance, which is not inhibited by ritonavir co-administration [14, 15]. Inhibition following this inhibitory I_max_ model is determined by ritonavir plasma concentrations, the inhibitory effect factor, KI, which was estimated to be 375 ng/mL (95% CI: 135–906), and the estimate of the maximum inhibitory effect (I_max_) of 570 L/h (95% CI: 400–776). Previous reports have indeed demonstrated that incubation of human liver microsomes with docetaxel in the presence of 1.8∙10^–3^ ng/mL ritonavir substantially inhibited formation of the M1/M3, M2, and M4 metabolites. On the other hand, when these microsomes were incubated with paclitaxel in the presence of ritonavir, the formation of 3’-p-hydroxy-paclitaxel, which is formed by CYP3A mediated oxidation, was completely inhibited, while formation of the 6α-hydroxy-paclitaxel remained relatively unaltered [13]. Finally, it was decided not to incorporate renal clearance in our population PK model given the reported minor contribution of this elimination route to total paclitaxel clearance [1].

The effect of the different oral paclitaxel formulations could be identified as covariates on gut bioavailability and the BETA parameter in the Weibull function. The relative gut bioavailability of the tablet and capsule formulations, as a fraction of the gut bioavailability of the drinking solution, was estimated to be 0.97 (95% CI: 0.67–1.33) and 0.46 (95% CI: 0.34–0.61), respectively. Despite its reasonable bioavailability, the drinking solution is not considered suitable for clinical use because of its unpleasant taste, high content of ethanol, and limited dosing accuracy.

The difference in gut bioavailability between the ASD formulations may be the result of the switch in the manufacturing method of the solid dispersion (from freeze-drying used in ModraPac capsules, to spray drying used in ModraPac tablets). Compared to freeze-drying, spray drying resulted in a solid dispersion with a higher and prolonged enhanced paclitaxel solubility. The explanation for that could be that spray drying resulted in a fully amorphous solid dispersion, whereas the freeze-dried solid dispersion was partially amorphous, due to recrystallization of SDS (included in the solid dispersion as a precipitation inhibitor) [17].

The different estimates for BETA resulted in distinct time-varying absorption rate profiles for the drinking solution and ASD formulations. The BETA_tablet/capsule_ of 3.57 (95% CI: 2.99–4.52) resulted in a more kurtotic time-varying absorption rate profile compared to the drinking solution (BETA_drinking solution,_ 2.53, 95% CI: 2.34–2.76). This difference is likely a result of the dissolution step required with the ASD formulations, while the drinking solution is administered in a dissolved state.

Addition of the empirical parameter *rF*_gut, 2nd/1st_ led to a considerable improvement of the model fit to the data, indicating a lower bioavailability of the second daily dose. Likely, this is a result of the increased gastric emptying time induced by ritonavir [13, 50]. This delayed gastric emptying time induces a delay in the absorption of the second daily dose, which is confirmed by the different estimates of ALPHA_1st daily dose_ and ALPHA_2nd daily dose_, resulting in a later T_max_ for the second daily dose, as shown in Supplementary figure S4. As a result of the increased gastric emptying time, a larger proportion of paclitaxel may have precipitated from super saturation, and would therefore no longer be available for absorption. Since PK was not sampled beyond 24 h in the LDM study, no data are available for this effect on subsequent days of LDM chemotherapy. Future studies using this formulation should therefore preferably incorporate PK sampling on subsequent days.

We successfully modelled the relationship between oral paclitaxel PK and TSP-1 upregulation in circulating platelets. Despite the fact that increases in TSP-1 levels were only observed in a few patients, inclusion of the PK/PD effect significantly improved the fit of the model to the TSP-1 observations. The established EC_50_ may serve as guidance for a PK target for potential future studies that investigate an exposure–response relationship between LDM with paclitaxel and TSP-1. The window of plasma concentrations attained using a LDM schedule with the RP2D (Fig. 4) are relatively low compared to the estimated EC_50_. Nevertheless, at steady state, paclitaxel plasma concentrations at this dose level are estimated to induce increases in TSP-1 formation rate (k_in_) ranging from 5.4% to 22.0%.

Based on the current performed PK simulations, comparison between IV and oral paclitaxel illustrated that LDM with the oral paclitaxel tablet formulation reaches AUCs in the range of conventional IV administration per 3 week interval, whereas C_max_ was substantially lower. Moreover, LDM treatment with tablet formulation demonstrated a longer T_C>0.05_ compared to the IV schedules. However, these PK parameters should be interpreted with caution. As mentioned above, the IV formulation of paclitaxel contains Cremophor EL, which forms micelles in the central circulation. Therefore, distribution may be limited after IV administration compared to oral administration [48]. Some studies have reported that the unbound paclitaxel concentration may be 2.6-fold higher after administration of a Cremophor EL-free formulation in comparison to a formulation that contains Cremophor EL [51].

## Conclusion

The complex PK of different oral paclitaxel formulations co-administered with ritonavir were adequately described with the developed PK model. Moreover, the developed model allowed for exploration of the pharmaceutical characteristics of three oral formulations. The spray dried ASD tablet formulation and drinking solution of paclitaxel showed the highest gut bioavailability. Simulations have shown that LDM with oral paclitaxel successfully achieves longer T_C>0.05_ compared to the IV formulations while retaining a comparable drug exposure to the standard IV paclitaxel regimens. Our PK/PD evaluation demonstrated a relationship between paclitaxel plasma concentrations following LDM treatment and TSP-1 upregulation with an EC_50_ of 284 ng/mL. Further research is necessary to strengthen this PD target and to shed more light on the overall clinical relevance of TSP-1 upregulation. In addition, investigation of the effect of ritonavir on gastric emptying time and oral paclitaxel bioavailability in case of repeated daily co-administration is warranted.

## Supplementary Information

Below is the link to the electronic supplementary material.

Supplementary file1 (DOCX 14 KB)

## Data Availability

The datasets analyzed during the current study are available from the corresponding author upon reasonable request.

## Code availability

The models described in the current study are available from the corresponding author upon reasonable request.
