# Supplementary material for: Development of a population pharmacokinetic/pharmacodynamic model for various oral paclitaxel formulations co-administered with ritonavir and thrombospondin-1 based on data from early phase clinical studies
Source: Cancer Chemother Pharmacol. 2022 Jul 7;90(1):71–82. doi: 10.1007/s00280-022-04445-z (PMC9300539; doi:10.1007/s00280-022-04445-z)
Supplement: Supplementary file 1 — Supplementary file1 (DOCX 14 KB) [file 280_2022_4445_MOESM1_ESM.docx]

| **Parameters** | **Units** | **Estimate** | **95% CI** | **Shrinkage (%)** |  |
| --- | --- | --- | --- | --- | --- |
| **Population parameters** |  |  |  |  |  |
| EC_50_ | ng/mL | 284 | 122 - 724 | - |  |
| E_BASE_ | ng/mL/10^6^ platelets | 43.8 | 39.7 – 48.5 | - |  |
| Turnover | h | 233 FIX | - | - |  |
| **Between-subject variability** |  |  |  |  |  |
| E_BASE_ | CV% | 28.2 | 22.8 – 36.6 | 4 |  |
| **Residual unexplained variability** |  |  |  |  |  |
| σ_prop_ | CV% | 13.8 | 12.3 – 15.8 | 12 |  |
|  | | | | | |
